# Optimizing evacuation efficiency under emergency with consideration of social fairness based on a cell transmission model

**DOI:** 10.1371/journal.pone.0207916

**Published:** 2018-11-27

**Authors:** Xuedong Yan, Xiaobing Liu, Yulei Song

**Affiliations:** MOE Key Laboratory for Urban Transportation Complex Systems Theory and Technology, School of Traffic and Transportation, Beijing Jiaotong University, Beijing, P. R. China; Shandong University of Science and Technology, CHINA

## Abstract

Traffic assignment and management objectives are considered as two significant parts in developing the emergency evacuation plan, which can directly influence the evacuation performance and efficiency. From the perspective of disaster response operators, the evacuation objective frequently is to minimize the total evacuation time to reduce losses, which may lead to an unreasonable and unfair phenomenon where people in highest risk areas may be forced to sacrifice their priorities of evacuation to improve the system evacuation efficiency. In this paper, considering both efficiency and social fairness in emergency evacuation, a weight function consisting of risk evaluation index as variable and the emphasis degree of managers on social fairness principle as coefficient was initially proposed and embedded in system optimal (SO) objective function. Combining the weight function and other constraints based on an extended cell transmission model (CTM), the linear program (LP) model was established to realize the simulation of dynamic traffic assignment in emergency evacuation. Employing this model, the impact of the management strategy of balancing both efficiency and social fairness on evacuation results was studied in the “Tianjin Explosions” case. In the end, the conclusion of “balancing social fairness is valuable during evacuation” was obtained.

## Introduction

When natural disasters, man-made accidents, terrorist attacks and other emergencies occur in urban areas, they may lead to huge losses of life and property. Once disaster events happen, how to effectively organize mass evacuation is of top priority for public management departments. Unfortunately, the previous studies, such as Ref. [1], have pointed out that there would be increasing difficulty and time consumption in mass evacuation with the increasing urban population and land-use density, as well as the growing road infrastructure. Severe large traffic congestions during disaster evacuation due to lacking valid evacuation plans were often reported [2, 3]. To prevent or at least minimize the damage caused by disasters, evacuating the suffered people to safe areas as efficient as possible is regarded as a main task for emergency management.

In general, how to systematically assign disaster victims into road network is the main body of making an evacuation plan, which could directly influence the evacuation efficiency and even the social stability in the disaster-affected areas. The dynamic traffic assignment (DTA) models have been utilized as useful tools for making evacuation plans [4–8], the theories of which can be roughly summarized as analytical models and simulation models. However, there are some obvious disadvantages for those models More specifically, most of the analytical models cannot reflect traffic realities well and the model solutions are difficult to caculatewhen a large-size or even middle-size road network is considered [9–12]. On the contrary, the simulation models can overcome these disadvantages, but essentially are myopic in evacuation route choice and fail to guaranteeing optimal solution [13–15]. Consequently, it is of vital significance for related scholars to formulate evacuation strategy and establish a theory model that can not only optimize dynamic traffic assignment, but also be well applied in large-scale regional road networks. Comparatively, the cell transmission model (CTM) is an innovative and efficient method to solve the DTA problem, which was first proposed by Daganzo [16, 17] as a discrete approximation of the hydrodynamic traffic flow model [18–20]. The CTM model can provide a simple solution method for analyzing road traffic conditions and accurately describe a series of traffic phenomenon in road network, such as the interference of traffic flow and the formation of shockwaves and link spillover.

As for the evacuation management objectives, the law-enforcement personnel often take the minimum evacuation time, clearance time and other travel time-based performance measures with the method of system optimal (SO) as the only target in most cases to improve evacuation efficiency. For instance, Ref. [21] used CTM to formulate a single destination system optimal dynamic traffic assignment model, which forms the basis of the various CTM-based evacuation planning models in the later literature [22–24]. Nevertheless, the evacuation objectives and decisions adopted by local disaster management agencies may differ, since diverse disasters have their own peculiarities. This type of traditional management strategy can sometimes make people in more dangerous areas suffer from serious damage due to the delay of traveling, which violate the ethical and equitable principles obviously. Therefore, it is necessary to consider both system efficiency and fairness as the determinants of evacuation management target. Some literatures indicated that evacuees show selfish behavior and tend to select routes that take them to the nearest shelter sites in a disaster situation [25–28]. Compromising both individual choice and system optimal, Ref. [29] proposed constrained system optimal (CSO) traffic assignment model to honor the travelers’ needs by imposing additional constraints to ensure that drivers are assigned to “acceptable” paths only. Ref. [30] considered fairness principle in evacuation by assigning evacuees to their neighbouring shelter sites and meanwhile minimized the total evacuation time as the evacuation target. However, the conception of fairness in the CSO model was based on the individual interests rather than social fairness and moral principles from the systematic perspective, the path allocation of which is static and determined by the constraint conditions of travelers’ initial locations.

In this paper, we developed a CTM-based LP model to simulate the allocations of evacuees in cell-bridge traffic network which can optimize evacuation efficiency in emergency management and meanwhile embody the social fairness. Compared to the existing methods, the proposed technique will make significant contributions from the fourfold aspects. (1) The CTM model was ameliorated and simplified in terms of traffic assignments among multiple bridges and bridge-based intersection control, which reduces the computational work and improves calculation efficiency in actual road network; (2) The location-based risk evaluation function was built and embedded into the evacuation management objective to quantify the social fairness and give residents different priorities in accordance with their risk levels; (3) The proposed risk evaluation function associated with a series of weighted coefficients is introduced to study the corresponding changes of evacuation performance for the whole system and the communities in different risk levels, which provided various kinds of management schemes to disaster response operators; (4) The Tianjin Explosions incident was used as an actual road network and emergent evacuation scenario to obtain the results of the dynamic traffic assignments under three types of objectives and some interesting findings were obtained.

The remaining part of this paper is arranged in the following order. Section 2 gives how to develop CTM model from the aspects of cell links and intersection control. The total risk cost function is established as evacuation target in section 3. The “Tianjin Explosions” incident taking place in China is utilized as an example for the model application in section 4. Finally, Section 5 provides some concluding remarks and the directions of subsequent research.

## CTM model development

### Fundamental theory

In the cell transmission model, Daganzo [16, 17] assumed that the relationship between traffic flow (*q*) and density (*k*) showing in Eq (1), which discretized the hydrodynamic traffic flow model in the spatial-temporal distribution and was proved to be computationally efficient and easy to analyze yet capture many important traffic phenomena. In addition, *v*, *q*_max_, *w* and *k*_*j*_ are constants and respectively denote the free flow speed, the maximum flow, backward wave speed and jam density.

$$q=\min\left\{vk,q_{\max},w(k_{j}-k)\right\},\;0\leq k\leq k_{j}$$ (1)

In the CTM, the time periods are discretized to the same small intervals and each road is consisted of many small homogeneous segments (named cells), the length of which is equal to the travel distance of vehicle driving at free flow velocity in one time interval. In this paper, the set of discrete time intervals and the set of cells respectively is denoted by *T* and *C*, and $x_{i}^{t}$ represents the number of vehicles in cell *i* at time *t*. For every cell, the following constants are defined: $N_{i}^{t}$ and $Q_{i}^{t}$ is respectively the maximum number of vehicles that can travel in one cell and can flow into or out of cell *i* at time *t*; $\delta_{i}^{t}$ is the ratio *v*/*w* of cell *i* at time *t*.

**Fig 1:**
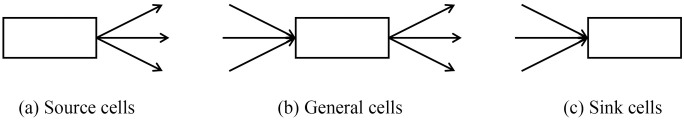
The classification of cells. (a) Source cells. (b) General cells. (c) Sink cells.

According to the topology structure of road network, adjacent cells are connected by “bridges”, which represent the relationship of traffic flow transmitting between adjacent cells. In this paper, the connected bridges are denoted with $y_{ij}^{t}$, namely the flow moving from cell *i* to cell *j* at time *t*, and the set of bridges is expressed as *B*. In addition, we define Γ^−1^(*i*) and Γ(*i*) as the sets of bridges that can flow into or out of cell *i* respectively. Above all, the state of the road network at any time *t* can be described by a series of variable $x_{i}^{t}$ and $y_{ij}^{t}$. In accordance with the value of Γ^−1^(*i*) and Γ(*i*), the set of cells *C* can be divided into three sub-sets: source cells *C*_R_(|Γ(*i*)|≥1, |Γ^−1^(*i*)| = 0), sink cells *C*_S_ (|Γ(*i*)| = 0, |Γ^−1^(*i*)|≥1) and general (merge-diverge) cells *C*_G_(|Γ(*i*)|≥1, |Γ^−1^(*i*)|≥1) as shown in Fig 1.

It should be pointed out that the source cells and the sink cells were presumed to only have one adjacent cell in Ref. [17], which ignored the existing of merge-diverge cells with more than one bridges linking upstream and downstream cells. It was inconsistent with the reality, especially for the complex networks of urban traffic.

### State transition equations and limitations of traffic transmission

Generally speaking, the cell transmission model is developed by two steps. The first is about cells: the state transition equations of each kind of cells were constructed. The second is about cell bridges: upper-bound values of all types of cell bridges were defined. Taking the initial evacuation demand transmitting from the source cells into consideration, the state transition equations of traffic flows among source cells, sink cells and other cells are established respectively in Eqs (2), (3) and (4), where $d_{i}^{t}$ is the evacuation demand at time *t* and in source cell *i*∈*C*_R_, while *X*_*i*_ is the number of vehicle in cell *i* at the beginning of evacuation.

$$x_{i}^{t}=x_{i}^{t-1}+\sum_{k\in \Gamma^{-1}(i)}y_{ki}^{t-1}-\sum_{j\in \Gamma (i)}y_{ij}^{t-1}\;\forall i\in C\backslash (C_{R}\cup C_{S})\;t\in T$$ (2)

$$x_{i}^{t}=x_{i}^{t-1}+d_{i}^{t-1}-\sum_{j\in \Gamma (i)}y_{ij}^{t-1}\;\forall i\in C_{R},\;t\in T$$ (3)

$$x_{i}^{t}=x_{i}^{t-1}+\sum_{k\in \Gamma^{-1}(i)}y_{ki}^{t-1}\;\forall i\in C_{S},\;t\in T$$ (4)

$$x_{i}^{0}=y_{ij}^{0}=0\;\forall i,j\in C$$ (5)

In Ref. [17] and Ref. [31], the set of connecting bridges was also partitioned into five sub-sets in accordance with the above classification of cells. In fact, the relationship of cells and connecting bridges in merge-diverge cells can be seen as general form in CTM model. Hence, in this paper, the value constrains of connecting bridges were simplified by focusing on the each cell itself rather than on the cells and bridges respectively. Considering the trapezoidal structural relationship between traffic flow and density in Fig 1 and the maximum number of vehicles $N_{i}^{t}$ within cells *i*, the constraint conditions of connecting bridges is listed in Eq (6) and Eq (7).

$$\sum_{k\in \Gamma^{-1}(i)}y_{ki}^{t}-Q_{i}^{t}\leq 0\;\sum_{k\in \Gamma^{-1}(i)}y_{ki}^{t}-\delta_{i}^{t}(N_{i}^{t}-x_{i}^{t})\leq 0\;\forall i\in C\backslash C_{R},t\in T$$ (6)

$$\sum_{j\in \Gamma (i)}y_{ij}^{t}-x_{i}^{t}\leq 0\;\sum_{j\in \Gamma (i)}y_{ij}^{t}-Q_{i}^{t}\leq 0\;\forall i\in C\backslash C_{S},t\in T$$ (7)

Eq (6) is the constrains of cell bridges flowing into cell *i* and Eq (7) is the constrains of cell bridges flowing out of cell *i*. Compared with Ref. [17] and Ref. [31], we would not concern traffic transmission assignments among multiple bridges any more but highlight the total traffic flow transmission from the perspective of each cell in order to improve simulation efficiency.

### Constraints on conflicting traffic flow at intersections

Existing works in evacuation planning typically considered free-flow models in which evacuees were dynamically routed in the network without constraining evacuees from a same area by a same path. In the application of CTM model, Ref. [21] was the first to incorporate CTM into the SO-DTA problem for a single destination network. The most of follow-up studies based on this model scheme conducted the extension of modeling traffic flow characteristics and dynamics during evacuation in a simple traffic network without conflicts, such as MCTM model proposed by Ref. [32] and SCTM model proposed by Ref. [33]. In many cases, however, majority of traffic delays during an evacuation occurred at intersections [34]. Considering the intersection control in evacuation planning, Ref. [25] suggested an alternative lane-based routing strategy to eliminate the crossing conflicts, which provided an effective and feasible approach to reduce traffic delays at intersections. At least two recent studies [35, 36] have introduced this intersection delay-reducing strategy into evacuation planning problems. Using CTM model to describe the road network, the influence of intersection (refers to the plane intersection in this paper) on traffic flow cannot be ignored, which is mainly manifested on the bifurcation, convergence and conflict of traffic flow. In fact, limitations of traffic flow transmission between adjacent cells shown as Eqs (6) and (7) have already covered the constrains of bifurcation and convergence on traffic flow. Subsequently, we would study about how the conflict points in intersection restrict traffic flow.

**Fig 2:**
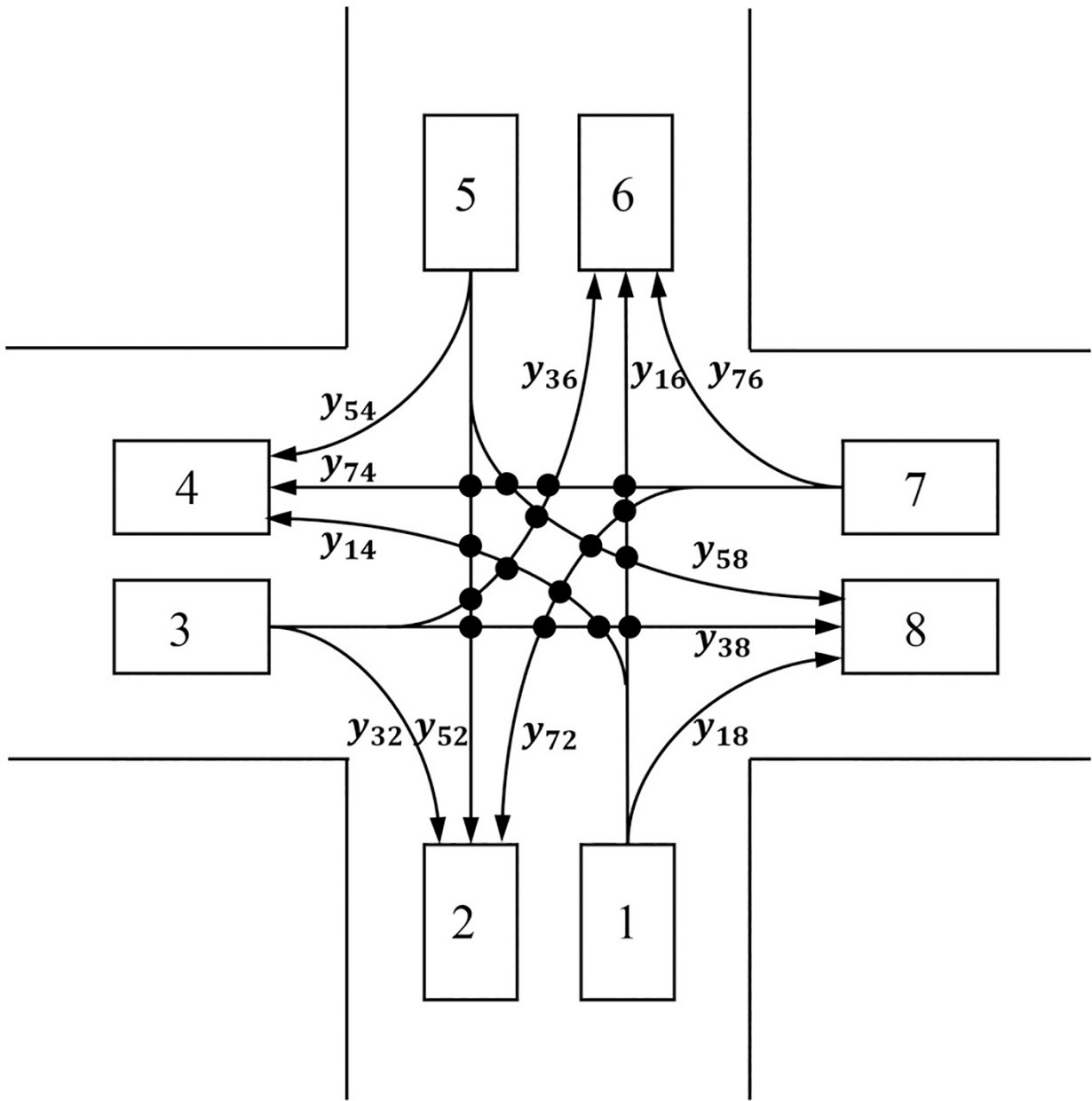
Conflicting bridge constraints in cross road intersection.

In this paper, any conflict of traffic flow between two linked cells is called conflicting bridge, and any pair of traffic flow conflicting with each other are denoted as mutual conflicting bridges. According to the lane-based routing method, we proposed the bridge-based control strategy as follows. The value ranges of conflicting bridge and mutual conflicting bridge are depended on the limitation of traffic capacity of intersections on road network. Firstly, total value of all conflicting bridges at the intersection must not exceed the maximum traffic capacity of intersection at any unit time; secondly, total value of any pair of mutual conflicting bridge at the intersection must not exceed a certain part of maximum traffic capacity of intersection at any unit time. A adjustment coefficient *α* is introduced to represent the value of certain proportion of maximum traffic capacity, and the smaller the value of *α*, the chance of conflicting is lower at intersections. Combining the above two aspects of constraints and manual control by law-enforcement personnel in emergency evacuation, the possibility of traffic flow conflicts at the intersection can be reduced, or even eliminated without the traffic lights control system. Taking cross road intersection as an example, there are 8 cells, 12 bridges, 8 conflicting bridges and 16 pairs of mutual conflicting bridges in Fig 2. Assuming maximum traffic capacity of intersection in any unit time is *Q* as shown in Eq (8), then the constraints can be denoted as Eq (9).

$$y_{14}+y_{16}+y_{36}+y_{38}+y_{52}+y_{58}+y_{72}+y_{74}\leq Q$$ (8)

$$\begin{array}{l} y_{16}+y_{38}\leq \alpha_{1}*Q;y_{16}+y_{58}\leq \alpha_{2}*Q;y_{16}+y_{72}\leq \alpha_{3}*Q;y_{16}+y_{74}\leq \alpha_{4}*Q;\\ y_{38}+y_{52}\leq \alpha_{5}*Q;y_{38}+y_{14}\leq \alpha_{6}*Q;y_{38}+y_{72}\leq \alpha_{7}*Q;y_{52}+y_{36}\leq \alpha_{8}*Q;\\ y_{52}+y_{14}\leq \alpha_{9}*Q;y_{52}+y_{74}\leq \alpha_{10}*Q;y_{74}+y_{36}\leq \alpha_{11}*Q;y_{74}+y_{58}\leq \alpha_{12}*Q;\\ y_{14}+y_{36}\leq \alpha_{13}*Q;y_{14}+y_{72}\leq \alpha_{14}*Q;y_{58}+y_{36}\leq \alpha_{15}*Q;y_{58}+y_{72}\leq \alpha_{16}*Q; \end{array}$$ (9)

In the road network based on CTM, the set of intersections with conflicting traffic flow is expressed as *M*. $W_{k}^{t}$ and $CB_{k}^{t}$ respectively represent the maximal traffic flow, the set of all conflicting bridges at intersection *k* among time *t*. Moreover, parameter $\alpha_{\phi }^{k}$ denotes the adjustment coefficient of maximal traffic flow for a certain mutual conflicting bridge pair at intersection *k*, where *ϕ* is the serial number of each mutual conflicting bridge pair. For any conflicting bridge $y_{ij}^{t}$, we define $B_{k}^{t}|y_{ij}^{t}$ as the set of bridges conflicting with $y_{ij}^{t}$ mutually. Therefore, the quantitative limitation of conflicting bridge at any intersection can be normatively denoted as Functions (10) and (11).

$$\sum_{y_{ij}^{t}\in B_{k}^{t}}y_{ij}^{t}\leq W_{k}^{t}\;i,j\in C;t\in T;k\in M$$ (10)

$$y_{ij}^{t}+y\leq \alpha_{\phi }^{k}*W_{k}^{t}\;0<\alpha_{\phi }^{k}<1;y\in B_{k}^{t}|y_{ij}^{t};y_{ij}^{t}\in B_{k}^{t};i,j\in C;t\in T;k\in M;$$ (11)

Eq (10) and Eq (11) respectively means the constraints of any conflicting bridge and mutual conflicting bridge on road network. According to both Eq (6) and Eq (7), the value range of each connecting bridge has been proposed, whether it is between cells or at intersections. Rather than using routine fixed signal control at intersections, the limitation of traffic flow conflicting was introduced to be a control strategy at intersection, which fully makes use of the intersection capacity and reduces the total time of emergence evacuation. It should be noted that this bridge-based control strategy was illustrated by the mathematical constraint of intersection capacity. As for the specific measures, managers may resort to uninterrupted flow facility provided by Ref. [25, 36] or other manual control projects.

## Evacuation objective function

With the SO-CTM evacuation plan from perspective of total evacuation time, the varying degrees of hazardousness during evacuation for people in different levels of danger zones might be neglected, which should be against ethical principles and evacuees’ social fairness. During evacuation, the social fairness means that the evacuation of people who are more vulnerable to disasters or in more dangerous conditions should not be delayed by evacuees with lower risk. In other words, the evacuation strategy should prioritize people in higher risk areas to leave out of danger, which was called Innermost-First-Out (InFO) control strategy, proposed by Ref. [37, 38]. According to InFO, the upstream freeway flows received priority over ramp flows and InFO control was optimal in light of two evacuation objectives: maximizing the number that could be evacuated at any time and minimizing the evacuation time. They argued that InFO could be socially acceptable as it prioritizes upstream residents who are likely to be more at risk. However, there was still lack of quantitative analysis for the InFO control strategy to grade priorities in accordance with risk levels.

In this paper, the evacuation management strategy compromising both the evacuation efficiency and social fairness was proposed to minimize the total hazard cost, which was defined as balancing fairness system optimal (short for BFSO). A hazard evaluation function for every cell in CTM model is proposed to identify the assistance needs quantitatively. For simplicity and with no loss of generality, three basic assumptions are made for this model.

1. The model would be only applicable to the cases of posterior evacuation after disaster occurrence and all residents in hazard influence area take cars.
2. The evacuation planning zone is well-defined by spatial impact scope of disasters, such as earthquake, explosion, toxicant leakage, and so on. Moreover, the evacuation planning zone’s risk level is in the relation of inverse proportion to the distance from disaster occurrence site to the zone.
3. The disaster response operators would guide the whole evacuation process and all residents should evacuate under the operators’ guidance.

In the previous hazards management literatures, the risk level of a location was primarily determined based on the potential disaster’s impact on that location relative to its geographical features, such as the distance of some community away from the location of disaster. The "Hazard scores" had been proposed to designate the risk level over the study region for planning purposes [39, 40]. In particular, Ref. [40] combined various geophysical risk factors into a hazard score as a multiplicative function of event frequency, scope (area), and intensity. Others have formulized the geophysical risk as a function of recurrence intervals [41, 42]. From the socioeconomic perspective, however, vulnerability was usually attributed to a human-induced situation resulting from public policy and resource availability. Some studies [43–45] had applied social vulnerability into the risk assessment to represent the level of needing evacuation assistance for communities.

In this paper, however, based on the assumptions 1 and 2, the evacuees' characteristics and resource availability would not be taken into account, because of the various types of disasters and the difficulty of obtaining actual data timely during the initial period of emergency. Instead, a risk evaluation function *R*(*d*_*i*_) shown in Eq (12) was proposed to denote the geophysical risk in a certain location according to the assumption 2, where *d*_*i*_ represents the distance of cell *i* from hazard installations. The risk evaluation function can quantitatively measure the spatial variation of risk that the evacuees in difference communities are exposed to during the evacuation. In fact, the risk evaluation function can reflect the dynamic assistance need for individual evacuee in each cell of road network at different time. In the CTM, the objective of SO evacuation strategy based on total evacuation time can be expressed as Function (13), where *τ* is the unit time interval. Taking minimal total risk cost of all residents during the whole evacuation procedure as part of management strategy, the evacuation objective was modified into Function (14).

$$R(d_{i})=1/d_{i}$$ (12)

$$Min\;\sum_{t\in T}\sum_{i\in C}\tau *x_{i}^{t}$$ (13)

$$Min\;\sum_{t\in T}\sum_{i\in C}R(d_{i})*x_{i}^{t}*\tau$$ (14)

Function (14) guarantees that the people who need more assistance or are in a more dangerous situation should drive away from their evacuation zones in advance through combining the risk evaluation function *R*(*d*_*i*_), which embodies the social fairness principle in the evacuation planning. Furthermore, a weight function *u*[*R*(*d*_*i*_),*θ*] was introduced to study the change of evacuation efficiency of the total system or the communities in different risk levels. The coefficient *θ* in the weight function reflects the degree of emphasis on social fairness in the management strategy. Before identifying the concrete form of weight function *u*[*R*(*d*_*i*_),*θ*], some function characteristics need to be proposed in accordance with the evacuation strategy and objective.

**Fig 3:**
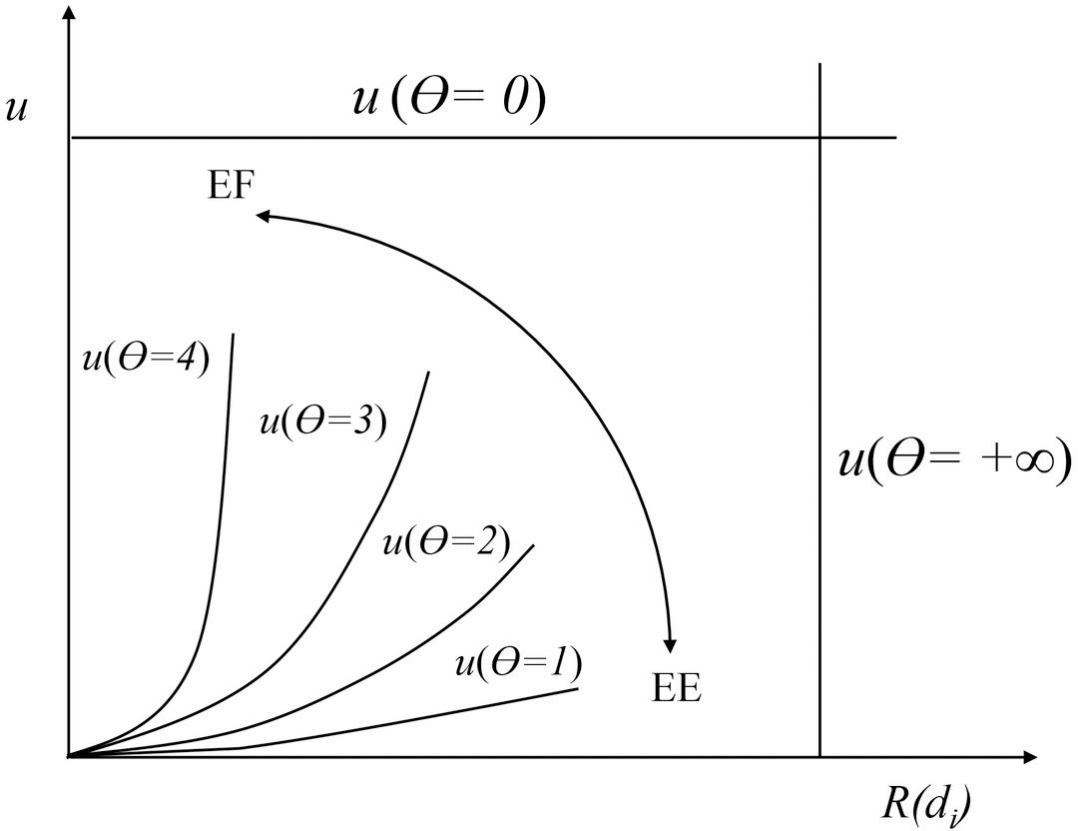
Weight function curves with different emphasis on efficiency and fairness.

1. *u*[*R*(*d*_*i*_),*θ*] is a monotone increasing function with *R*(*d*_*i*_) as variable and *θ* as coefficient.
2. *θ* = 0 and *u*[*R*(*d*_*i*_),*θ*] = 1 denotes *u*[*R*(*d*_*i*_),*θ*] would not influence management strategy and objective anymore; *θ* = 1*u*[*R*(*d*_*i*_),*θ*] = *R*(*d*_*i*_) is equivalent to Function (14); if *θ* = +∞, *u*[*R*(*d*_*i*_),*θ*] will also be infinite.
3. The value of *θ* can directly decide the degree of emphasis on efficiency or social fairness. In Fig 3, a series of weight function curves with variation of coefficient *θ* reflect a set of weights, where EF and EE denote the trend of emphasizing fairness and efficiency respectively. The steeper the below curve is, the objective function more focuses on social fairness in evacuation.

To the best of my knowledge, there was no practical case setting social fairness principles as evacuation management objective from the system perspective. Therefore, there is no available historical data to derive the exact expression form of the weight function by fitting method. Considering the variation trend of weight function curves with different coefficients, one of basic elementary functions, general power function, was chosen as the weight function in this paper, which could well satisfy the aforementioned function characteristics. Therefore, the weight function was presented in Eq (15) with the coefficient *θ* as the power. We can not prove the power form of the weighted risk function proposed in this work is the optimal one, but there is no doubt that this weighted risk function is the suitable one in actual evacuation management according to the function characteristics.

$$u\left[R(d_{i}),\theta \right]={\left[R(d_{i})\right]}^{\theta }$$ (15)

In accordance with Eq (15), the *θ* decides the steep degree of weight function curve and how much the planners emphasize the social fairness or efficiency in evacuation. If *θ* is down to zero, i.e. *u*(*θ* = 0), the objective only focus on the evacuation efficiency, namely perfect efficiency system optimum (PESO) management strategy; if *θ* is growing to infinity, i.e. *u*(*θ* = +∞), the objective is a perfect fairness system optimum (PFSO). From all above discussion, the final evacuation objective was proposed considering both social fairness principle and efficiency through embedding weight function *u*[*R*(*d*_*i*_),*θ*] into PESO management strategy, shown as Function (16):

$$Min\;\sum_{t\in T}\sum_{i\in C}{\left[R(d_{i})\right]}^{\theta }*x_{i}^{t}*\tau \;\theta \geq 0$$ (16)

In summary, combining the weight Function (16) compromising both efficiency and social fairness and the following constraints of CTM model, the LP model is proposed in this work.

$$\left\{ \begin{array}{l} x_{i}^{t}-x_{i}^{t-1}-\sum_{k\in \Gamma^{-1}(i)}y_{ki}^{t-1}+\sum_{j\in \Gamma (i)}y_{ij}^{t-1}=0\;\forall i\in C\backslash C_{R},\;t\in T\\ x_{i}^{t}-x_{i}^{t-1}-d_{i}^{t-1}+\sum_{j\in \Gamma (i)}y_{ij}^{t-1}=0\;\forall i\in C_{R},\;t\in T\\ x_{i}^{t}=x_{i}^{t-1}+\sum_{k\in \Gamma^{-1}(i)}y_{ki}^{t-1}\;\forall i\in C_{S},\;t\in T\\ x_{i}^{0}=X_{i}\;\forall i\in C,\;t\in T \end{array}\right.$$ (17)

$$\left\{ \begin{array}{l} \sum_{k\in \Gamma^{-1}(i)}y_{ki}^{t}-Q_{i}^{t}\leq 0\;\sum_{k\in \Gamma^{-1}(i)}y_{ki}^{t}-\delta_{i}^{t}(N_{i}{}^{t}-x_{i}^{t})\leq 0\;\forall i\in C\backslash C_{R},t\in T\\ \sum_{j\in \Gamma (i)}y_{ij}^{t}-x_{i}^{t}\leq 0\;\sum_{j\in \Gamma (i)}y_{ij}^{t}-Q_{i}^{t}\leq 0\;\forall i\in C\backslash C_{S},t\in T\\ \sum_{y_{ij}^{t}\in B_{k}^{t}}y_{ij}^{t}\leq W_{k}^{t}\;i,j\in C;t\in T;k\in M\\ y_{ij}^{t}+y\leq \alpha_{\phi }^{k}*W_{k}^{t}\;0<\alpha_{\phi }^{k}<1;y\in B_{k}^{t}|y_{ij}^{t};y_{ij}^{t}\in B_{k}^{t};i,j\in C;t\in T;k\in M \end{array}\right.$$ (18)

$$x_{i}^{t},y_{ij}^{t},y_{ki}^{t}\geq 0\;k\in \Gamma^{-1}(i),j\in \Gamma (i);\;\forall i\in C,t\in T$$ (19)

The constrains Eq (17) are the state transmission equations between each cell including the initial value of each cell on road network. The constrains Eq (18) limit the value ranges that the general bridges and conflicting bridges can be assigned. The constrains Eq (19) are the nonnegative restriction of variables. Thus, the CTM-based LP model with the objective of balancing both efficiency and social fairness has been completely presented. An interior-point algorithm was adopted to resolve the linear programming model, which possesses a higher efficiency for working out the large and complex linear programming issue.

## Numerical example Study-Tianjin explosions

In this section, the Tianjin Explosions incident was used as an actual road network and emergent evacuation scenario to: a) compare the evacuation results under two objective functions i.e., minimize total evacuation time and minimize total weighted evacuation time considering social fairness; b) obtain the results of above LP model including the dynamic traffic assignments.

### Description for Tianjin explosions

On August 12, 2015, at least two explosions within 30 seconds occurred at a container storage station at the Port of Tianjin in the Binhai New Area of Tianjin, China, which caused 173 deaths, 8 missing and 798 non-fatal injuries. In the blasts, many chemicals leaked, such as the sodium cyanide that can react with water to form highly toxic and flammable hydrogen cyanide gas. Considering the secondary explosions and toxic substances spreading risks, the local authority issued an evacuation order that required people in dangerous areas (within 3 kilometers to the explosions site) to evacuate from their houses. The disaster impact scope was regarded as organized-evacuation region or evacuation planning zone. According to the Chinese National Standard, i.e. Operation Conditions and Technical Requirements for Enterprises Handling Dangerous Chemicals Business (GB 18265–2000), large and medium-sized dangerous chemicals warehouses shall be at least 1km away from surrounding public buildings, major transportation lines (roads, railways, waterways) and industrial and mining enterprises. Therefore, the range of 1 km radius are considered extremely dangerous area and are necessary to delimit it specially in this paper. In addition, the main fatalities, most of whom were rescue workers, happened in radius of 200m centered the exploration site, which is not the evacuation object in this paper. As usual, efficiency was still top consideration in this evacuation for decision makers, without considering the social fairness from the system perspective.

### Descriptions for road network and evacuation demand

**Fig 4:**
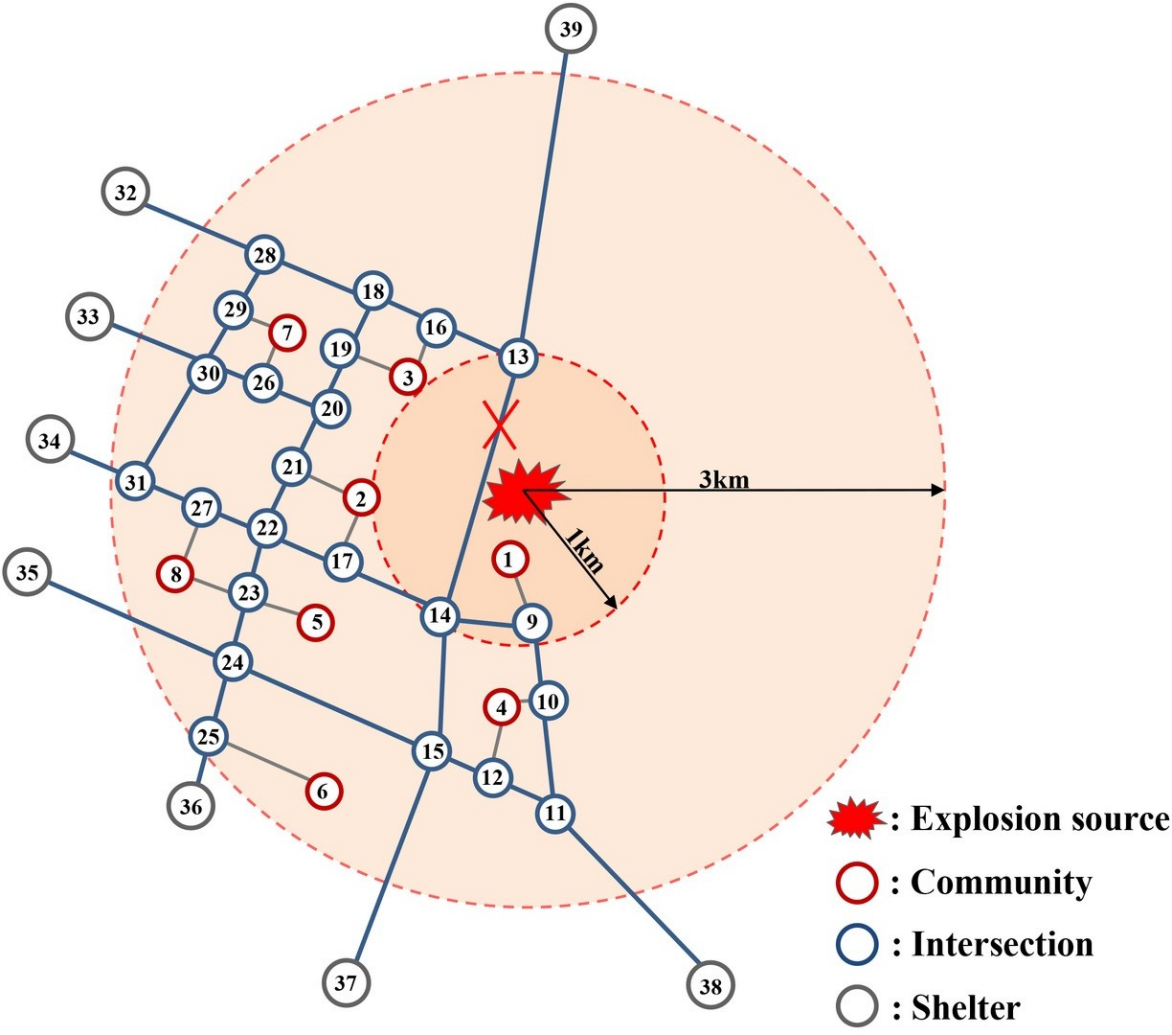
The node-arc representation for road network of the organized-evacuation area.

As is shown in the map Fig 4, there were 8 regrouped communities labeled with numbers from 1 to 8 according to the sequence of distance to the explosion site in the evacuation area. The community number's coding principle is that the smaller the numbers, the closer the communities' distances to the explosion source are. There were totally about 36000 residents from above 8 communities living in the organized-evacuation areas, who needed to be evacuated to the shelters as soon as possible after the evacuation order was issued. It should be noted that the junctions of evacuation road network and safe area boundary could be regarded as fictitious shelter locations, since the evacuees must go through the fictitious shelters in order to reach the safe havens. To be more specific, nodes 1 to 8 are communities that need to be evacuated; the nodes 32 to 37 represent fictitious shelters outside the government evacuation region; the other nodes denote intersections in road network. According to the assumption in CTM model, evacuees must take the nearest intersections as origin to drive away from communities. In addition, road segment between node 13 and 14 was cut off because of the explosions.

In this example, the time interval *τ* was set as 10 seconds. There is lack of the specific population distribution data to determine the evacuation demand of every community. Therefore, it was assumed there would be 4500 residents needed to leave in each community and they all chose private cars as traffic mode. However, the validity and advantage of CTM-based LP model considering both efficiency and social fairness proposed in this paper is still working when actual data are available and input. If each car could carry an average of 3 persons, the evacuation demand of every community would be 1500 vehicles and the total demand would be 12000 vehicles.

### Cell-bridge structure of the road network

**Fig 5:**
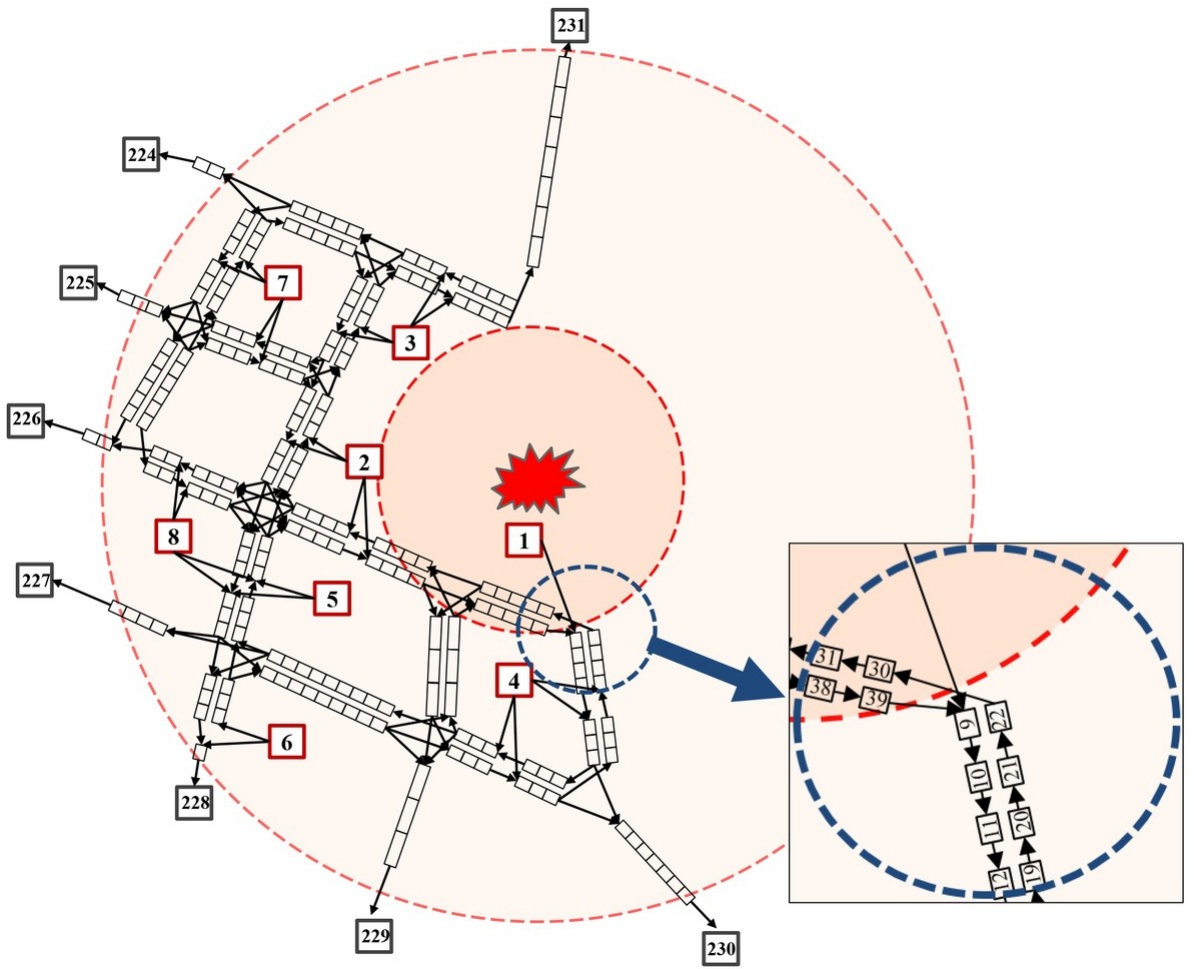
The cell-bridge structure of the road network in evacuation.

The particular conditions of the road network and the cells in this case are outlined in Table 1. The speed limit and the maximum traffic flow refer to Chinese Industrial Standards, Urban of Road Design Code (cjj37-2012), while the number of lanes and road length are measured by the electronic map and the ranging tools embedded in the map. The speed limits in general roads and highways were 60km/h and 120km/h respectively. Similarly, the maximum number of vehicles for each cell was determined by the size of the cell and the number of lanes. The radio $\delta_{i}^{t}$ was set as 1 no matter in any time interval or any cell. In addition, the maximum traffic flow of each intersection is determined by the modified maximum capacity of road segment connected with them. With the assumption and data above, the whole road network can be clearly described by the cell-bridge structure in Fig 5. There are totally 231 cells (including 8 source cells and 8 sink cells) and 282 connection bridges in this CTM road network.

**Table 1:** The attributes of the example network and the cells.

Road	13–32	20–33	9–34	11–35	9–11	37–39	18–36	28–31
Length (km)	2.38	1.17	3.35	2.75	1.75	3.04	3.68	2.00
No. of Lanes	3	3	4	3	3	4	4	3
Speed Limit (km/h)	60	60	60	60	60	120	60	60
Max Flow (vph)	5400	5400	7200	5400	5400	8560	7200	5400
Length of Per Cell (km)	0.167	0.167	0.167	0.167	0.167	0.334	0.167	0.167
No. of Cells	27	27	36	27	36	72	36	27
Max No. of Vehicles in cells	13–32	20–33	9–34	11–35	9–11	37–38	18–36	28–31

Considering the change of the property of concavity and convexity for the weight function when coefficient 0<*θ*<1 or *θ*>1, we should pick coefficients in these two interval, respectively. Besides, objective function is a power function with rigid monotone increasing characteristic, so a series of power values (*θ* = 0, 0.5, 1, 2, 3, inf) were taken among above two intervals to analyze and compare the influences of various management strategies on systematic and partial efficiency. It should be noted that the value of $\alpha_{\phi }^{k}$ (adjustment coefficient of maximal traffic flow) all employ 0.5 in this case, i.e. the maximum capacity of intersection in a certain direction, which could try to avoid the conflicting traffic flow without corroding the evacuation capacity at intersection.

### Results analyses

The solution of this model consisted of two steps. The first step is to calculate the weight coefficient for each cell with different power *θ* values in the weight function; the second is to calculate the status value of each cell and bridge respectively under certain power *θ* value. The simulation calculation is by Lingo11.0, which can solve the large-scale linear programming problem with fast solution speed and high stability. The traffic assignment results were given out under a series of weight coefficients (*θ* values) at every moment. The total evacuation time *T*_*E*_ (the sum of evacuation time of every vehicle) and the clearance time *T*_*C*_ (the time from the first car’s evacuation to the last car’s arrival at shelters) of both a certain community and the whole region were recorded to evaluate the evacuation efficiency.

For the sake of discussion and analysis, the 8 communities were divided into two classes according to their distances to explosion, namely high-risk communities (near explosions site, more dangerous) and low-risk communities (far away from explosions site, less dangerous). The high-risk ones were the communities 1, 2, 3 and 4 while the low-risk ones were the communities 5, 6, 7 and 8. The evacuation times of all four high-risk communities would be given and studied specifically. Residents in high-risk communities are more vulnerable to the disaster and their trips should be prioritized, so we focus on the time-based evacuation performance of both the high-risk communities and whole region under various evacuation objectives.

#### Analyses on efficiency and social fairness in evacuation

In this case, evacuation efficiency was denoted by total evacuation time and clearance time while social fairness was represented by the increase of evacuation efficiency for high-risk communities along with the value increment of coefficient *θ*. Table 2 recorded the total evacuation time and clearance time of both whole region and high-risk communities under different evacuation objectives, i.e. different weight coefficient values (*θ* = 0, 0.5, 1, 2, 3, inf).

**Table 2:** Total evacuation time and clearance time in the region and high-risk communities.

Objective	Time	High-risk communities				Whole region
		1	2	3	4	
PESO(*θ* = 0)	*T*_*E*_ (10^5^s)	8.11	8.49	8.30	9.99	72.02
	*T*_*C*_ (s)	1130	1120	1120	1100	1150
BFSO(*θ* = 0.5)	*T*_*E*_ (10^5^s)	5.42	3.77	3.71	6.91	72.04
	*T*_*C*_ (s)	610	470	520	730	1170
BFSO(*θ* = 1)	*T*_*E*_ (10^5^s)	5.42	3.75	3.69	6.91	72.15
	*T*_*C*_ (s)	600	450	520	730	1200
BFSO(*θ* = 2)	*T*_*E*_ (10^5^s)	5.34	3.64	3.61	6.86	72.83
	*T*_*C*_ (s)	600	450	500	730	1280
BFSO(*θ* = 3)	*T*_*E*_ (10^5^s)	5.24	3.57	3.53	6.88	74.26
	*T*_*C*_ (s)	600	450	490	740	1350
PFSO(*θ* = 4)	*T*_*E*_ (10^5^s)	5.24	3.57	3.51	6.91	82.50
	*T*_*C*_ (s)	600	450	480	740	1540

**Fig 6:**
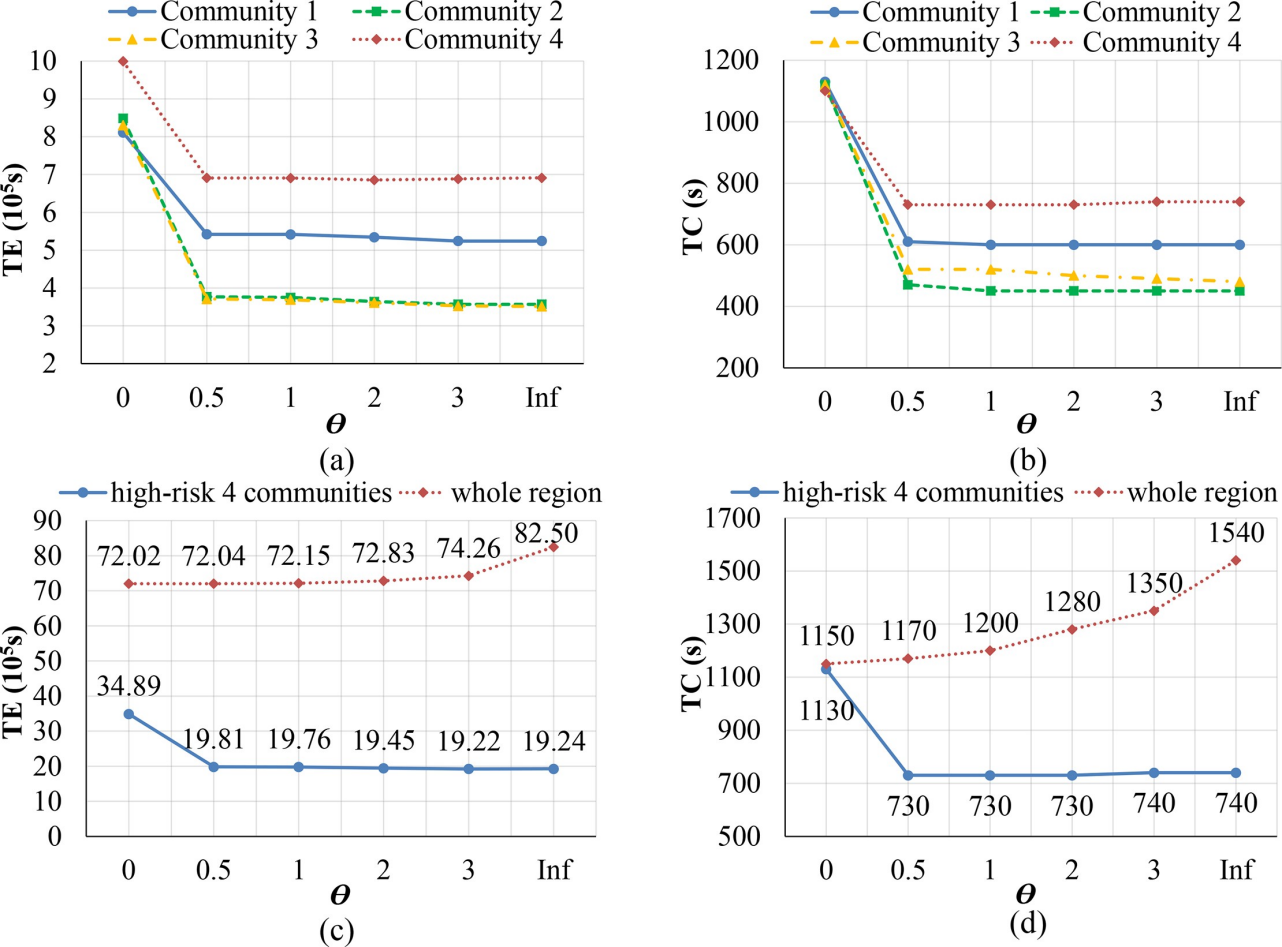
Evacuation efficiency of whole region and high-risk communities with *ϴ* varying. (a) *T*_*E*_ in different high-risk communities. (b) *T*_*C*_ in different high-risk communities. (c) The sum of *T*_*E*_ in high-risk communities and whole region. (d) *T*_*C*_ in high-risk communities and whole region.

It can be observed easily that along with the weight coefficient values *θ* increasing, evacuation objective focuses on social fairness more and more in this model. Therefore, the total evacuation time and clearance time in high-risk communities show a downward trend, but they are all gradually rising from the point of whole region. When *θ*>5, the evacuation performance of high-risk communities would not change any more. Therefore, using four various weighted coefficients of BFSO are sufficient to analyze the sensitivity of the optimal solutions. Fig 6 described the changes in detail, where the clearance time in high-risk communities was presented by the maximum clearance time among the four communities. Fig 6(A) and 6(B) respectively show the changes of two type of time indicators for every high-risk community, while Fig 6(C) and 6(D) describe the sums of evacuation time and clearance time from the perspective of all the four high-risk communities and whole region.

As can be seen from Fig 6(A) and 6(B), once the impact of social fairness principle is added into the model objective, namely *θ* = 0 was changed into *θ* = 0.5, the total evacuation time and clearance time of each high-risk community have an evident decline trend. Nevertheless, if *θ* keeps increasing, the downward trend of *T*_*E*_ and *T*_*C*_ becomes slow and even invariable, or even a little ascending in Community 4. The main cause is that once *θ*>0, weight function starts taking effect and immediately enhances the priority of each high-risk community in evacuation. Hence, their evacuation performance is greatly impacted. Along with *θ* going up continuously, Community 1, 2, and 3 will fall down slowly until to the minimum time values. However, there is a modest upward trend for the total evacuation time and clearance time of Community 4 since *θ*≥2, which is probably because that Community 2 has the furthest distance to hazard and lowest danger level among four high-risk communities, namely medium-risk community. Therefore, the increase of *θ* more stresses road usage priority of other three high-risk communities, and thus delays the evacuation of residents in Community 4.

From the Fig 6(C) and 6(D), it can be found that the total evacuation time and clearance time of four high-risk communities are also sharply cut down once the weight coefficient *θ* is changed into *θ* = 0.5. If *θ* keeps going up, both the time values decrease slowly and mostly keep constant. On the contrary, the total evacuation time and clearance time of the whole region are rising gently in the beginning, then increasing quickly along with *θ* turned into Inf, namely regarding PFSO as objective. Specifically, when *θ* = 0 is changed into *θ* = 0.5, the total evacuation time of four high-risk communities is reduced from 34.89*10^5^s to 19.81*10^5^s, about a 43.2% reduction, and their clearance time is dropped about a 35.4% reduction. Meanwhile, the total evacuation time of the whole region is rising from 72.02*10^5^s to 72.04*10^5^s, only 0.03% up, and their clearance time is added 1.7% up. This indicates while the evacuation objective is BFSO (when *θ* = 0.5 or *θ* = 1) rather than PESO (when *θ* = 0), the evacuation efficiency of high-risk communities is improved sharply with the cost of barely sacrificing evacuation efficiency of the whole region.

However, if the evacuation objective is turned into PFSO, i.e. *θ* = Inf, compared with *θ* = 0.5, the total evacuation time of four high-risk communities is reduced only 2.9% and their clearance time is even a little upward owing to the evacuation delay in community 2, but both the time of whole region correspondingly are increased by 14.5% and 31.6%. It indicates while the evacuation objective become PFSO rather than BFSO, the evacuation efficiency for high-risk communities has little change, but the system evacuation efficiency and the rights and interests of people in low-risk communities are impacted a lot. According to above analysis, a crucial conclusion in evacuation is proposed, namely “balancing social fairness is valuable during evacuation”. This conclusion means that when the PESO evacuation management strategy becoming BFSO, namely embodying the social fairness principle in evacuation plan, the system evacuation efficiency is only slightly decreased, but the efficiencies of high-risk communities are enhanced greatly, which is worth employing during evacuation. However, when the evacuation objective is totally turned into PFSO, the evacuation efficiencies of high-risk communities cannot be further improved while the evacuation efficiency of the whole region would be greatly reduced. Considering the evacuation objective taking weighted cells as basic spatial unit according to the risk levels, people living in high-risk level communities would always evacuate in priority with limited evacuation capacity of road network. Therefore, the various initial demand distribution in evacuation may have an impact on picking the more suitable coefficients for the evacuation objective of balancing fairness system optimal (BFSO), but it would not change the conclusion of balancing social fairness is valuable during evacuation.

### Dynamic traffic assignment results

Taking weight coefficient *θ* = 1 as an example, where the plan objective can be regarded as minimizing total risk cost, a series of dynamic traffic assignment results were given and total evacuation time *T*_*E*_ and clearance time *T*_*C*_ in the whole region and each community were shown as the Table 3.

**Table 3:** The total evacuation time and clearance time in the whole region and communities.

Community No.	1	2	3	4	5	6	7	8	total
Total evacuation time (10^5^s)	5.42	3.75	3.69	6.91	10.68	13.88	8.90	18.92	72.15
clearance time (s)	600	450	520	730	870	1100	910	1200	1200

When the weight coefficient *θ* = 1, the total evacuation time is 72.15*10^5^s and clearance time is 1200s from the perspective of whole region. High-risk Communities 1, 2, 3, and 4 spend less time to complete evacuation, among which Community 3 takes the least total evacuation time and Community 2 needs the least clearance time. These low-risk Communities 5, 6, 7, and 8, in contrast, need more time to evacuate and Community 8 needs the maximum. The main reason of this phenomenon is that we endowed the high-risk communities with the priority to evacuating from dangerous area through emphasizing on evacuation social fairness in the objective function, so the high-risk communities spent less total evacuation and clearance time than those communities far away from the explosion sites. However, both the total evacuation time and clearance time are not strictly sequenced according to the dangerous level of each community. For example, Community 1 is closest to the hazard and under most dangerous condition, but its total evacuation time and clearance time are longer than Community 2, which may be due to the special road network topology structure in organized-evacuation area. As shown in the Fig 6, Community 2 can choose more bridges (with four bridges while Community1 only has two bridges) to transmit evacuation vehicles, so the evacuation of Community 2 was faster than Community 1. The case of Community 6 is similar with Community 1.

**Fig 7:**
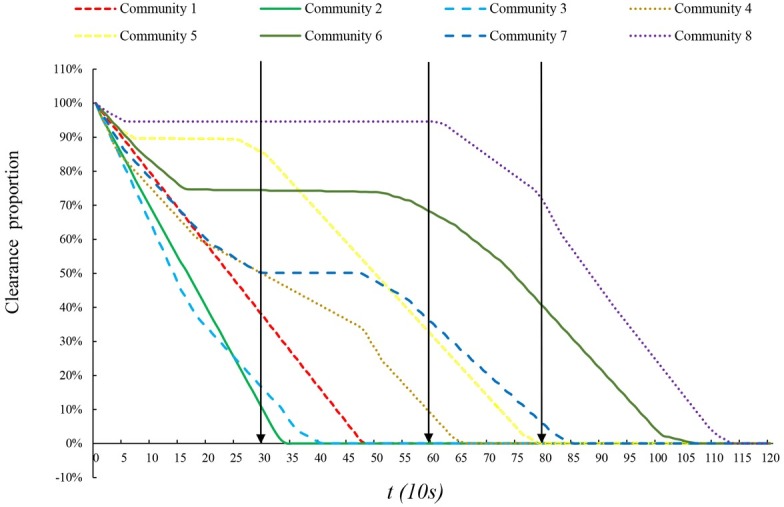
Proportion of rest vehicles to evacuate in each community during evacuation.

Because the clearance time in evacuation for the whole region is 1200s and unit time is 10s, there is 120 unit time to conduct the traffic distribution. Thus, the dynamic traffic assignment results should be composed by all the assignment states in per unit time. In this paper, every assignment state during 120 unit time was recorded and then the continuity of assignment state during certain period was identified. Without considering the travel time of evacuees in road network after departing from residences, the evacuation process of each community is shown as Fig 7. In the beginning of evacuation, every community started moving out. Then along with the first teams of evacuees’ continually travelling in planning evacuation region, people in low-risk communities stopped evacuating temporarily to yield to the evacuees from high-risk communities, which shows the social fairness principle in management strategy. Along with the reduction of risk level, the evacuation delay time of low-risk communities shows a rising trend, but Community 7 is out of this pattern and delay less time. It may relate to its location and traffic condition in road network. In the other hand, traffic management personnel can implement the control counseling programs at the exits of each community according to the specific evacuation pause time for low-risk communities simply and effectively. After the high-risk communities finished evacuation, people living in low-risk communities would not delay again, but evacuate sparing no effort until all people left from their houses.

**Fig 8:**
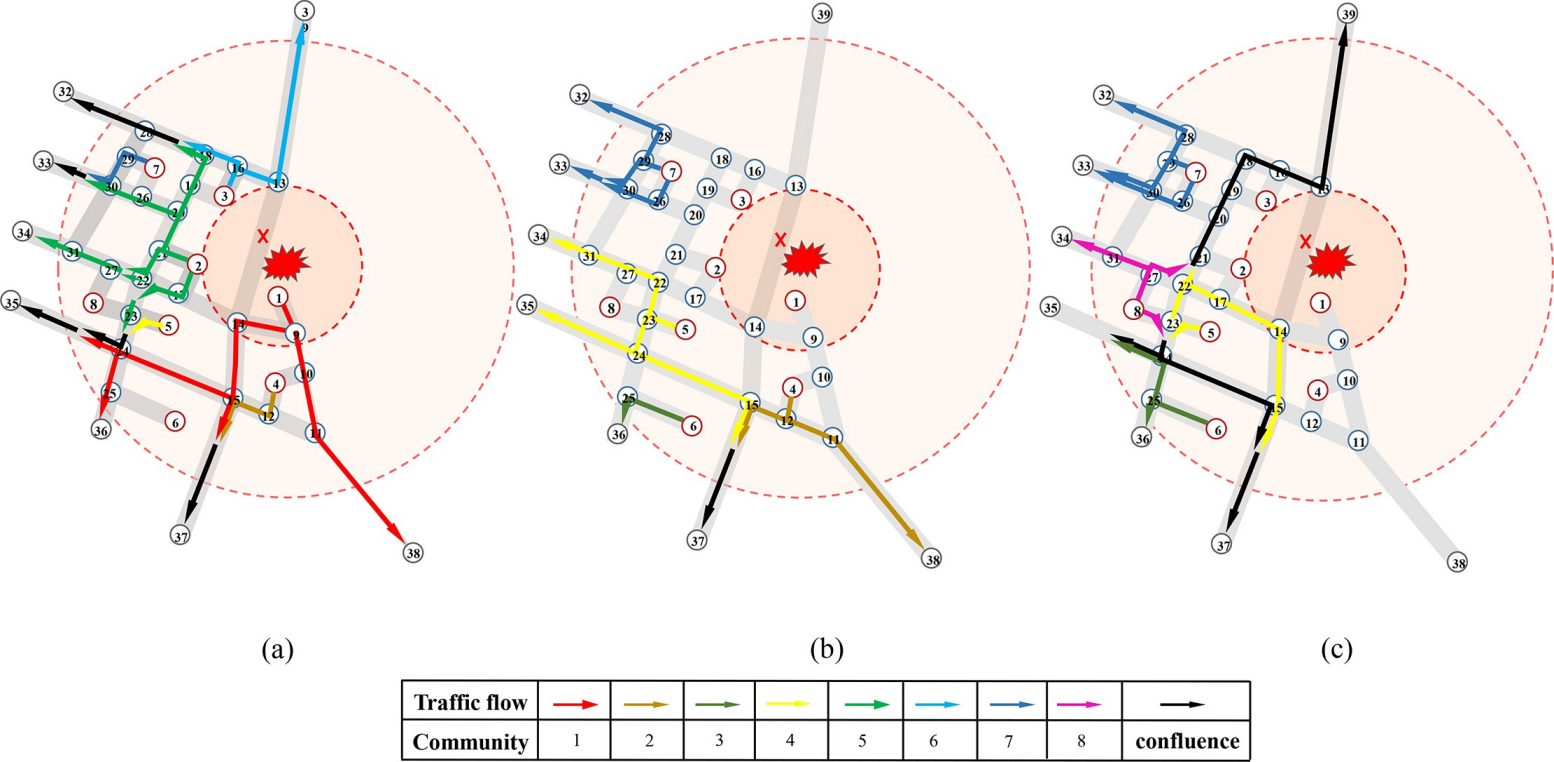
Path distribution results of communities at three significant time nodes. **(a)** Path distribution results of communities at t = 30. **(b)** Path distribution results of communities at t = 60. **(c)** Path distribution results of communities at t = 80.

According to the clearance time in Table 3, all the assignment states can be basically described by three assignment states respectively in time *t* = 30 (no community finish evacuation), *t* = 60 (Communities 1, 2 and 3 have finished evacuation), *t* = 80 (Communities 1, 2, 3 and 4, namely all high-risk communities, have finished evacuation), which are marked by arrow lines in Fig 8. The dynamic traffic assignment results in the whole road network was described as follows:

When time *t* = 30 shown as Fig 8(A), people lived in Communities 1, 2, 3, 4, 5, 7 all started evacuation, among which Community 1 was arranged with four traffic flow paths (the red arrow lines), including path 1-9-10-11-38, path 1-9-14-15-24-35, path 1-9-14-15-24-36, and path 1-9-14-15-37. Community 2 occupied the most paths (the green arrow lines) to drive out, almost using all the routes that did not conflict with the evacuation of Community 1. Limited by the evacuation paths occupied by above communities and their own traffic conditions, Communities 4, 5, and 7 have only one evacuation path, while people in Communities 6 and 8 had not yet been evacuated. In addition, there was no conflicting point for all the intersections in the road network at time *t* = 30.

When time *t* = 60 revealed as Fig 8(B), people lived in Communities 1, 2, and 3 had finished the evacuation. Community 6 started to leave through path 6-25-36 (the army green arrow lines), attributed to Community 1 no longer occupying this path. Similarly, for other communities, the available paths were also increased to accelerate the whole evacuation, such as adding another path 4-12-11-38 to Community 4 and allocating path 5-23-22-31-34 and 5-23-24-15-37 to Community 5, but Community 8 had no one evacuated yet. Once again, there was no conflicting point for all the intersections at this time.

When time *t* = 80 shown as Fig 8(C), high-risk Communities 1, 2, 3 and 4 all ended evacuation. Compared with Path distribution results in time *t* = 60, Community 4 no longer occupied the road resource and Community 8 began to leave through paths 8-27-31-34, 8-27-22-18-13-39, 8-23-24-35, and 8-23-24-15-37 (the purple arrow lines). The path distribution results of other communities were also changed, such as Community 6 evacuating via additional path 6-25-24-35 at this moment. Similarly, there was no conflicting point for all the intersections.

In conclusion, there was no conflicting traffic flow in intersections at above three critical timing when $\alpha_{\phi }^{k}$ equal to 0.5, which proofed the validity of the constraints of conflicting traffic flow based on bridge proposed in the previous sections. Moreover, we do not have to loosen the restraints of mutual conflicting traffic flows by improving the adjustment coefficient, because it has guaranteed the maximum traffic capacity for one certain direction at intersection.

## Discussion and conclusion

Emergency evacuation management is becoming increasingly critical with the rise of both natural and man-made disasters. Dynamic traffic assignments and evacuation management objective are considered as two significant parts in making emergency evacuation plan, which can directly influence the evacuation performance and efficiency. In this paper, the indices including total evacuation time and clearance time were adopted to measure the evacuation performance and a risk level function was constructed to reflect the social fairness in the evacuation. From the perspective of evacuation management authority, a CTM-based LP model for simulating the dynamic traffic assignment considering both efficiency and social fairness in emergency evacuation was proposed.

Compared with the evacuation models in previous studies, there are some advantages and innovations in this study. Firstly, a CTM model considering both the different types of cell bridges and the bridge-based control strategy at intersections was adopted in the LP model, which can not only simplify the difficulty of the DTA issue but also reflect traffic realities well. Secondly, the social fairness is not negligible any more, to which is worth paying attention in the practical evacuation process. A risk evaluation function was proposed to denote the hazard cost in traffic network based on CTM model, which can guarantee people under more dangerous conditions having priority to evacuate dynamically. Thirdly, a weight function with risk evaluation function as variable and the emphasis degree of managers on social fairness principle as power was initially proposed and embedded in SO objective function, through which the evacuation managers can attach more or less importance to social fairness principle in the procedure of evacuation guidance according to their demand. Although some assumptions are not perfectly realistic in an evacuation context, they can form a reasonable approximation especially when enforcement personnel are actively involved in the implementation of the evacuation plan. Moreover, the proposed weighted risk evaluation function considering both efficiency and social fairness would influence the evacuation performance, which can provide various kinds of evacuation schemes to disaster response operators.

“Tianjin Explosions” was used as a numerical example to analyze the results of this model and a crucial conclusion “balancing social fairness is valuable during evacuation” was obtained. This conclusion means that when the model objective is shifted from only focusing on evacuation efficiency to considering both evacuation efficiency and social fairness, the evacuation time of the whole region is decreased slightly while the evacuation efficiency of the high-risk communities is improved sharply. If the power of the weight function continues to grow until the evacuation objective becoming PFSO, however, the efficiency of the high-risk communities will keep almost invariant along with a great loss of the evacuation efficiency of the whole region. In addition, there are apparent pause time periods for low-risk communities in the process of dynamic traffic assignment in evacuation, according to which traffic management personnel can implement the control counseling programs simply and effectively.

## Limitation and prospect

It should be pointed out that there were still some limitations and unaccomplished work in this paper. First, the location-based risk evaluation function proposed in this work is only applicable to the evacuation planning zone with well-defined spatial impact scope of disasters, where the risk levels of residents mainly hinge on their locations to disaster sites. The impacts of the characteristics of disaster, evacuee, and evacuation operations on social fairness principle are worth the follow-up study. Still taking the “Tianjin Explosions” case as example, if accurate data about population characteristic and resource allocation could be collected, it is necessary to integrate both geophysical risk (e.g. disaster characteristics, environmental factors) and social vulnerability (e.g. population composition, access to resources, and the level of social networks) to assess the overall evacuation assistance needs in certain locations and propose specific probability distributions of risk evacuation functions based on various kinds of evacuees for other types of disasters.

Moreover, the power form of the weighted risk function is proposed based on some particular values and variation trend of weight function curves, the optimality of which can not be proved. If the data from practical case considering social fairness during emergency evacuation is generated and extracted, then we can seek sufficient theoretical or empirical support or opposition to the proposed model. In contrast with the specific evacuation results including evacuation time and assignment route of each community under SO management strategy, we may provide more insights by applying the model to simulate the same evacuation scenario.

Another interesting extension is studying what perturbations will cause on the evacuation results when we consider the phenomenon of individual choice not following guidance system or police control. Introducing the data of personal preference into the source cells in CTM model may be a feasible solution. The dynamic traffic assignment results without conflicting points for all the intersections also demonstrated the validity of bridge-based control strategies to some degree. However, there are still lack of sufficient theoretical and empirical support for the bridge-based control strategies and optimal values of adjustment coefficient, which need to be further studied. Moreover, the evacuation model proposed in this paper has not discussed the subsequent evacuation after people arriving the fictitious shelters, which may be another interesting research topic.

## Supporting information

S1 Appendix Specific locations of all cells in road network of numerical example were shown in Table A and Figure A. (PDF)
