# Supplementary material for: Optimizing evacuation efficiency under emergency with consideration of social fairness based on a cell transmission model
Source: PLoS One. 2018 Nov 27;13(11):e0207916. doi: 10.1371/journal.pone.0207916 (PMC6258532; doi:10.1371/journal.pone.0207916)
Supplement: S1 Appendix — (PDF) [file pone.0207916.s001.pdf]

S1 Appendix

Table A is the specific location of all 231 cells in the CTM road network of the numerical example of the "Tianjin Explosions" based on the coordinate system in Figure A. The location of explosions is set as the origin of coordinate.

Table A. Specific location of all cells in road network

Cell Number	Distance along x-axis (km)	Distance along y-axis (km)
1	0	-0.3
2	-0.2	-1.2
3	-1.5	-2
4	-1.8	-0.9
5	-1.3	0
6	-0.9	1
7	-1.9	1.2
8	-0.2	-2.5
9	0.1	-1.1
10	0.1	-1.2
11	0.1	-1.4
12	0.1	-1.5
13	0.1	-1.7
14	0.1	-1.8
15	0.1	-2
16	0.1	-2
17	0.1	-1.8
18	0.1	-1.7
19	0.1	-1.5
20	0.1	-1.4
21	0.1	-1.2
22	0.1	-1.1
23	0.2	-2.2
24	0.3	-2.3
25	0.4	-2.4
26	0.5	-2.5
27	0.6	-2.6
28	0.7	-2.7
29	0.8	-2.8
30	0	-0.9
31	-0.2	-0.9
32	-0.3	-0.9
33	-0.4	-0.8
34	-0.6	-0.8
35	-0.6	-0.8
36	-0.4	-0.8

37	-0.3	-0.9
38	-0.2	-0.9
39	0	-0.9
40	0	-2.1
41	-0.2	-2
42	-0.3	-2
43	-0.4	-1.9
44	-0.5	-1.9
45	-0.7	-1.8
46	-0.7	-1.8
47	-0.5	-1.9
48	-0.4	-1.9
49	-0.3	-2
50	-0.2	-2
51	0	-2.1
52	-0.9	-2.1
53	-1	-2.3
54	-1	-2.6
55	-0.7	-1.1
56	-0.8	-1.3
57	-0.8	-1.6
58	-0.8	-1.6
59	-0.8	-1.3
60	-0.7	-1.1
61	-0.1	1.3
62	0	1.5
63	0	1.8
64	0.1	2
65	0.1	2.3
66	0.1	2.5
67	0.2	2.8
68	-0.9	-1.7
69	-1.1	-1.7
70	-1.2	-1.6
71	-1.4	-1.5
72	-1.5	-1.5
73	-1.6	-1.4
74	-1.8	-1.3
75	-1.9	-1.2
76	-2.1	-1.2
77	-2.1	-1.2
78	-1.9	-1.2
79	-1.8	-1.3
80	-1.6	-1.4

81	-1.5	-1.5
82	-1.4	-1.5
83	-1.2	-1.6
84	-1.1	-1.7
85	-0.9	-1.7
86	-2.2	-1.2
87	-2.2	-1.3
88	-2.3	-1.5
89	-2.3	-1.6
90	-2.3	-1.6
91	-2.3	-1.5
92	-2.2	-1.3
93	-2.2	-1.2
94	-2.4	-1.8
95	-1.9	-0.3
96	-2	-0.5
97	-2	-0.6
98	-2.1	-0.7
99	-2.1	-0.8
100	-2.2	-1
101	-2.2	-1
102	-2.1	-0.8
103	-2.1	-0.7
104	-2	-0.6
105	-2	-0.5
106	-1.9	-0.3
107	-2.4	-0.9
108	-2.5	-0.7
109	-2.7	-0.4
110	-2.8	-0.2
111	-0.8	-0.7
112	-1	-0.7
113	-1.1	-0.6
114	-1.2	-0.5
115	-1.4	-0.5
116	-1.5	-0.4
117	-1.6	-0.3
118	-1.8	-0.3
119	-1.8	-0.3
120	-1.6	-0.3
121	-1.5	-0.4
122	-1.4	-0.5
123	-1.2	-0.5
124	-1.1	-0.6

125	-1	-0.7
126	-0.8	-0.7
127	-2	-0.2
128	-2.2	-0.1
129	-2.3	-0.1
130	-2.4	0
131	-2.6	0.1
132	-2.6	0.1
133	-2.4	0
134	-2.3	-0.1
135	-2.2	-0.1
136	-2	-0.2
137	-2.8	0.1
138	-2.9	0.2
139	-1.6	0.6
140	-1.6	0.4
141	-1.7	0.3
142	-1.7	0.2
143	-1.8	0.1
144	-1.8	-0.1
145	-1.8	-0.1
146	-1.8	0.1
147	-1.7	0.2
148	-1.7	0.3
149	-1.6	0.4
150	-1.6	0.6
151	-2.4	0.9
152	-2.4	0.7
153	-2.5	0.6
154	-2.5	0.5
155	-2.6	0.4
156	-2.6	0.2
157	-2.6	0.2
158	-2.6	0.4
159	-2.5	0.5
160	-2.5	0.6
161	-2.4	0.7
162	-2.4	0.9
163	-1.6	0.8
164	-1.8	0.8
165	-1.9	0.9
166	-2	0.9
167	-2.2	1
168	-2.2	1

169	-2	0.9
170	-1.9	0.9
171	-1.8	0.8
172	-1.6	0.8
173	-2.4	1.1
174	-2.5	1.1
175	-2.6	1.2
176	-1.2	1.4
177	-1.2	1.2
178	-1.3	1.1
179	-1.4	1
180	-1.4	0.8
181	-1.4	0.8
182	-1.4	1
183	-1.3	1.1
184	-1.2	1.2
185	-1.2	1.4
186	-0.2	1.1
187	-0.4	1.1
188	-0.5	1.2
189	-0.6	1.3
190	-0.7	1.3
191	-0.9	1.4
192	-1	1.4
193	-1	1.4
194	-0.9	1.4
195	-0.7	1.3
196	-0.6	1.3
197	-0.5	1.2
198	-0.4	1.1
199	-0.2	1.1
200	-1.2	1.6
201	-1.4	1.6
202	-1.5	1.7
203	-1.6	1.8
204	-1.8	1.8
205	-1.8	1.8
206	-1.6	1.8
207	-1.5	1.7
208	-1.4	1.6
209	-1.2	1.6
210	-2	1.8
211	-2	1.6
212	-2.1	1.5

213	-2.1	1.4
214	-2.2	1.3
215	-2.2	1.1
216	-2.2	1.1
217	-2.2	1.3
218	-2.1	1.4
219	-2.1	1.5
220	-2	1.6
221	-2	1.8
222	-2	1.9
223	-2.1	2
224	-2.2	2
225	-2.7	1.2
226	-3	0.2
227	-3	0
228	-2.4	-1.9
229	-1.1	-2.8
230	0.9	-2.9
231	0.2	3

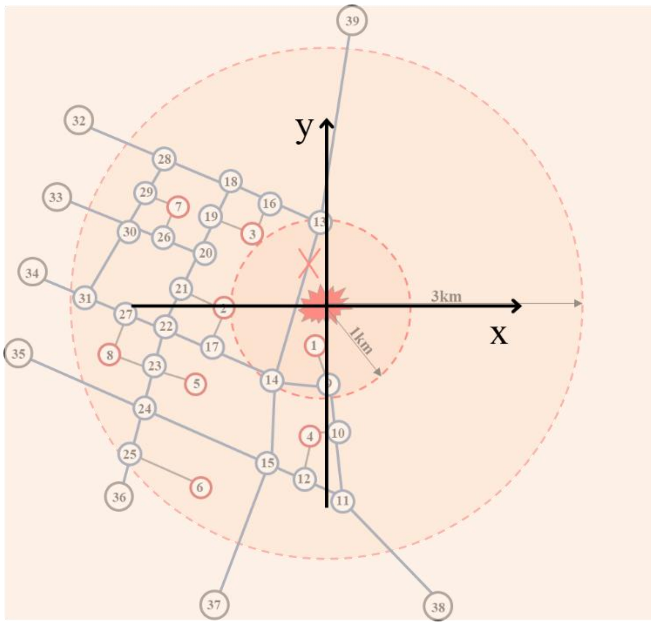

Figure A. The coordinate system of cells location in road network
